# Exploring new horizons in CAR-based therapy for the treatment of thyroid-associated ophthalmopathy

**DOI:** 10.1186/s40779-025-00590-7

**Published:** 2025-01-28

**Authors:** Xin-Yu Zhu, Wei-Yi Zhou, Tuo Li

**Affiliations:** Department of Endocrinology, the Second Affiliated Hospital of Naval Medical University, Shanghai, 20003 China

**Keywords:** Thyroid-associated ophthalmopathy (TAO), Graves’ ophthalmopathy (GO), Chimeric antigen receptor (CAR)-based therapy

Dear Editor,

Thyroid-associated ophthalmopathy (TAO), or Graves’ ophthalmopathy (GO), is a complex autoimmune condition characterized by eye symptoms such as proptosis, lid retraction, and periorbital swelling, often associated with thyroid dysfunction [1]. Current drug treatments primarily include glucocorticoids, traditional immunosuppressants, and novel biologics such as Teprotumumab. While able to alleviate symptoms, they often do not adequately address irreversible conditions such as visual impairment and fibrosis, and it is difficult to avoid long-term medication side effects. In this context, exploring new therapeutic avenues for TAO to improve patients’ prognosis and quality of life is meaningful. We proposed chimeric antigen receptor (CAR)-based therapy as a novel treatment orientation.

When TAO becomes severe, it can lead to serious complications such as dysthyroid optic neuropathy, vision loss, corneal ulceration, and fibrosis, significantly impairing patients’ quality of life. Intravenous glucocorticoids (IVGCs) are recommended as the first-line treatment for moderate-to-severe active TAO, aiming to reduce inflammation [2]. However, it has little impact on disease progression and tissue reconstruction, while accompanying the inevitable toxicity of glucocorticoids. Similarly, immunosuppressants like mycophenolate mofetil (MMF) and novel biologics such as Teprotumumab also show limited efficacy and are associated with considerable side effects.

CAR-based therapy has become a new pillar in treating autoimmune diseases (ADs). Based on the principles of CAR-based therapy, if a disease’s treatment strategy involves eliminating a specific type of cells and they can be specifically identified (i.e., targeted), then CAR-based therapy could serve as an effective approach for treating the disease. Attempts to apply CAR-based therapies in ADs have already begun, and TAO is no exception—it too presents a compelling opportunity for the exploration of CAR-based therapies as a potential innovative treatment. However, the key to success, also the premise, lies in selecting the most appropriate “weapon” to hit the TAO at its core. The most significant part of the “weapon” is identifying the right target, selected from the pathogenesis of TAO. Selected targets should provide broad coverage to ensure therapeutic efficiency while maintaining specificity to preserve safety [3].

The autoimmune response in TAO is complex, involving orbital fibroblasts (OFs), T cells, B cells, and a range of cytokines [4]. T and B lymphocytes infiltrate from the peripheral circulation into orbital tissues, beginning with recognizing thyroid-stimulating hormone receptor (TSHR) or insulin-like growth factor-1 receptor (IGF-1R). Among them, T cells which are considered as the predominant link differentiate into T helper (Th)1, Th2, and Th17 subsets, secreting cytokines that drive inflammation. They are seen to be a better target to curtail or eliminate abnormal immune responses from origin. CD19, CD20, B-cell activating factor receptor, and B cell maturation antigen have proven to be effective and popular targets in CAR-based trials for depleting B cells in ADs. Meanwhile, CD5 and CD7 are considered typical targets for eliminating T cells with autoimmune disorders. In addition, CD34^+^ fibrocytes migrate into the orbital environment, differentiating into OFs. These OFs further differentiate into myofibroblasts and adipocytes, and also accumulate hyaluronic acid, leading to orbital tissue expansion. So, targeting fibroblasts directly is possible to improve the bad outcomes closely related to clinical manifestation to some extent. CD34 may serve as a specific target for the selective elimination of OFs. However, because CD34 is widely expressed in stem cells, targeting it alone may cause substantial off-target effects, risking a “pyrrhic victory” where the harm to healthy tissues outweighs the therapeutic benefits. Given the complexity of disease pathogenesis in TAO and other ADs, it is challenging to identify an optimal target that balances broad efficacy with safety. To overcome these obstacles, advanced strategies can be leveraged to equip our CAR weapons.

Dual-target CAR-based therapy enhances specificity by targeting multiple surface antigens. For instance, TSHR and IGF-1R could be regarded as co-targets with other specific antigens on the cell surface in a CAR-based therapy, like locking CD34^+^ OFs through CD34 and TSHR. Off-the-shelf CAR cells, pre-manufactured from healthy donors, offer quicker and more affordable treatment options. It has already been explored in a clinical trial for myositis and systemic sclerosis, showing promising results [5]. Furthermore, the suicide gene strategy, which involves incorporating genes that allow CAR cells to be selectively eliminated in case of adverse effects, could add an extra layer of safety to these therapies. Further research is needed to establish and verify the clinical efficacy of CAR-based cell therapy in TAO and to explore additional innovative treatment strategies.

In conclusion, once the CAR is constructed after target selection and strategy equipment, its efficacy can be explored through in vitro and in vivo studies, followed by clinical trials (Fig. 1). These stages will help assess the CAR-based therapy’s potential to target and eliminate specific immune cells in TAO while evaluating its safety, efficacy, and potential side effects. We are hopeful that, through continued research, CAR-based therapy may prove to be a promising and effective treatment for TAO to provide a meaningful solution for patients facing severe and refractory cases of TAO.

**Fig. 1:**
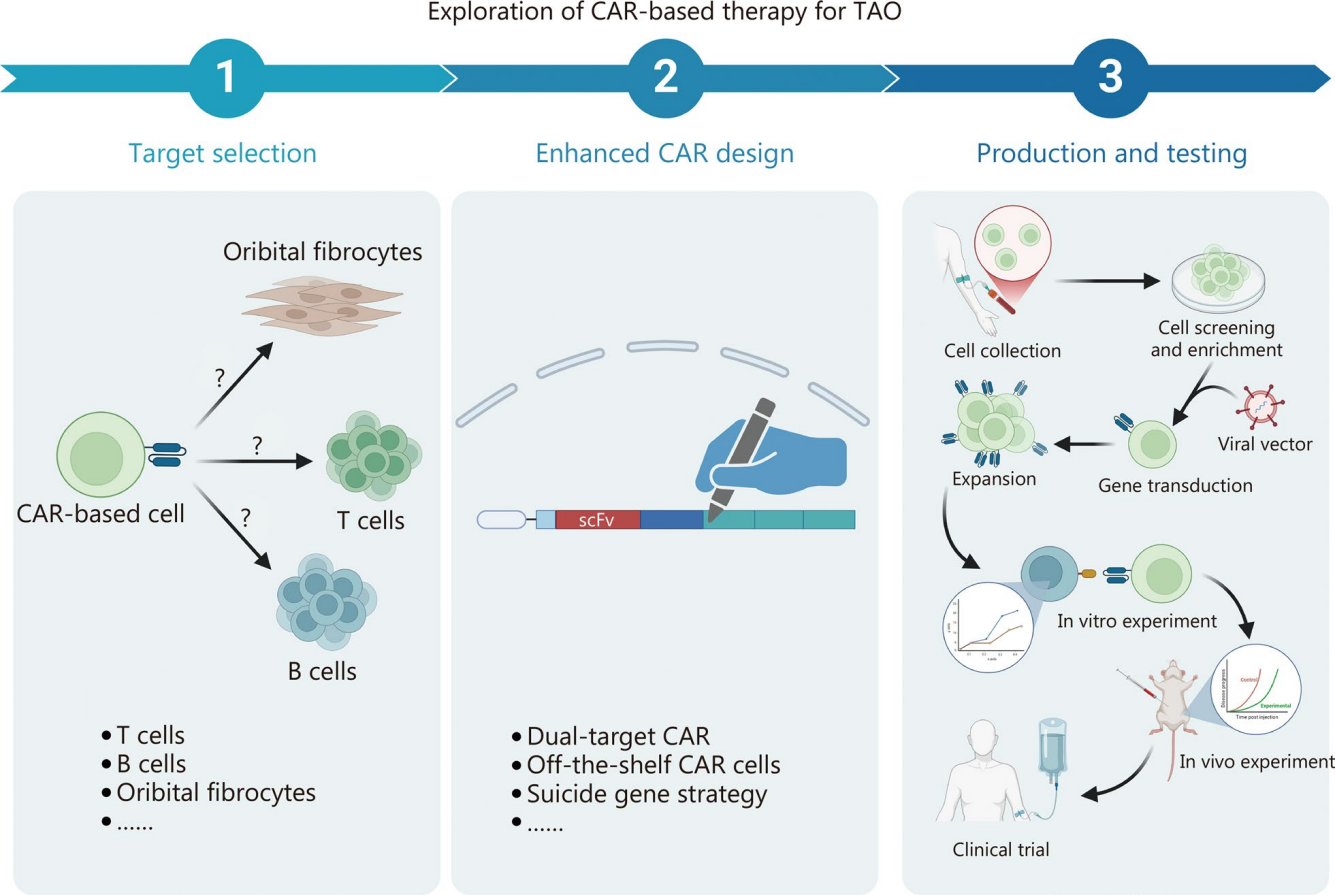
Exploration of chimeric antigen receptor (CAR)-based therapy for thyroid-associated ophthalmopathy (TAO). The process of exploring CAR-based therapy as a novel strategy for TAO can be divided into 3 sessions: target selection, enhanced CAR design, and production and testing. The initial step involves selecting an optimal target based on the pathogenesis of TAO, followed by employing innovative strategies to design the CAR sequence. Subsequently, after conducting successful in vitro experiments, participant-origin CAR immune cells can be utilized for clinical trials

## Data Availability

Not applicable.

## Abbreviations

ADs: Autoimmune diseases
CAR: Chimeric antigen receptor
GO: Graves’ ophthalmopathy
IGF-1R: Insulin-like growth factor-1 receptor
IVGCs: Intravenous glucocorticoids
MMF: Mycophenolate mofetil
OFs: Orbital fibroblasts
TAO: Thyroid-associated ophthalmopathy
Th1: T helper 1
Th17: T helper 17
Th2: T helper 2
TSHR: Thyroid-stimulating hormone receptor

## References

[1] Bahn RS. Graves’ ophthalmopathy. N Engl J Med. 2010;362(8):726–38. 20181974 10.1056/NEJMra0905750 PMC3902010

[2] Bartalena L, Kahaly GJ, Baldeschi L, Dayan CM, Eckstein A, Marcocci C, et al. The 2021 European Group on Graves’ orbitopathy (EUGOGO) clinical practice guidelines for the medical management of Graves’ orbitopathy. Eur J Endocrinol. 2021;185(4):G43-67. 34297684 10.1530/EJE-21-0479

[3] Wei J, Han X, Bo J, Han W. Target selection for CAR-T therapy. J Hematol Oncol. 2019;12(1):62. 31221182 10.1186/s13045-019-0758-x PMC6587237

[4] Chang HH, Wu SB, Tsai CC. A review of pathophysiology and therapeutic strategies targeting TGF-β in Graves’ ophthalmopathy. Cells. 2024;13(17):1493. 39273063 10.3390/cells13171493 PMC11393989

[5] Wang X, Wu X, Tan B, Zhu L, Zhang Y, Lin L, et al. Allogeneic CD19-targeted CAR-T therapy in patients with severe myositis and systemic sclerosis. Cell. 2024;187(18):4890-904.e9. 39013470 10.1016/j.cell.2024.06.027

